# Application of mind mapping combined with case-based learning model using simulated clinical cases in nursing education

**DOI:** 10.3389/fmed.2026.1754462

**Published:** 2026-03-06

**Authors:** Xia Niu, Xiaoxia Ku

**Affiliations:** Medical College Jinzhou Medical University, Jinzhou, Liaoning, China

**Keywords:** case-based learning, clinical thinking, mind mapping, nursing education, student satisfaction, teaching model

## Abstract

**Objective:**

This study aimed to evaluate the effectiveness of a mind mapping-integrated case-based learning (CBL) model using simulated clinical cases in nursing education.

**Methods:**

One hundred nursing undergraduates from a medical college were enrolled between January 2022 and January 2024. According to the teaching approach, students were assigned to either a control group receiving traditional lecture-based teaching or a research group receiving the mind mapping-integrated CBL model using simulated clinical cases (*n* = 50 per group). The two groups were compared in terms of theoretical examination scores, practical nursing skills scores, clinical thinking ability (including critical, evidence-based, and systematic thinking), learning initiative, and satisfaction with teaching following the intervention.

**Results:**

Following the intervention, students in the research group achieved significantly higher theoretical knowledge and practical nursing skill scores compared with those in the control group (*z* = 7.565 and 7.680, respectively; *P* < 0.05). In terms of cognitive outcomes, the research group demonstrated superior performance in critical, evidence-based, and systematic thinking relative to the control group (*z* = 4.149, 4.525, and 4.181, respectively; *P* < 0.05). In addition, significant improvements were observed in self-directed learning ability, learning motivation, and clinical application of knowledge in the research group (*z* = 4.651, 5.396, 6.032, respectively; *P* < 0.05). Overall satisfaction with the teaching method was also significantly higher in the research group than in the control group (*χ^2^* = 8.274, *P* < 0.05).

**Conclusion:**

The mind mapping-integrated CBL model using simulated clinical cases can markedly enhance nursing students’ theoretical mastery, clinical operational skills, and reflective thinking ability, while also improving satisfaction with the teaching process.

## Introduction

The basic nursing course is widely regarded as the cornerstone of undergraduate nursing education and serves as a key platform for cultivating essential professional competencies (1). Although this discipline covers a broad range of clinical content, persistent challenges remain in current teaching practice. Previous studies have reported that nursing students often exhibit inadequate academic preparedness, unclear learning motivation, limited learning autonomy, and inefficient use of extracurricular time (2–4). Despite these issues, instructional approaches in many higher education institutions continue to rely predominantly on teacher-centered knowledge transmission (5). Furthermore, the lack of systematic tools to evaluate students’ after-class review and knowledge consolidation restricts educators’ ability to tailor instructional strategies to individual learning progress. Consequently, students frequently struggle to effectively master course content, while instructors face difficulties in adjusting teaching methods to meet learners’ needs (2, 6). This dual challenge highlights the urgent need for evidence-informed teaching approaches that can simultaneously address deficiencies in both teaching and learning.

Practical skills training constitutes a core component of the basic nursing curriculum, accounting for approximately 50%–60% of total instructional hours (7). Traditionally, a “demonstration–practice–feedback” model has been widely adopted, emphasizing the imitation of standardized procedures following instructor demonstration (8). While this approach can facilitate the acquisition of procedural skills, it often limits opportunities for analytical thinking, critical reflection, and collaborative learning, thereby constraining students’ ability to translate theoretical knowledge into authentic clinical practice (9). Recognition of these limitations has prompted growing interest in alternative instructional strategies aimed at enhancing students’ reasoning, judgment, and problem-solving abilities (10).

Mind mapping is grounded in constructivist learning theory, which conceptualizes learning as an active and constructive process (11). By integrating visual elements with textual information, mind mapping establishes associative links between key concepts and images, colors, or symbols, thereby emphasizing the hierarchical structure and core elements of learning content. This process facilitates efficient information organization, storage, and retrieval (12). Empirical evidence suggests that mind mapping can promote the integration of fragmented knowledge, enhance analytical and problem-solving skills, and strengthen learners’ motivation and engagement (10, 12). Case-based learning (CBL) represents another effective pedagogical approach for creating authentic learning contexts and bridging theoretical knowledge with clinical practice (13, 14). Through autonomous and collaborative learning, students analyze clinical cases, discuss potential solutions, and apply foundational knowledge to practical scenarios (15). It has been demonstrated that CBL can effectively enhance nursing students’ theoretical understanding, clinical reasoning, independence, and problem-solving capacity (15).

From a theoretical perspective, the integration of mind mapping and CBL exhibits strong complementarity and coherence. CBL provides meaningful clinical scenarios for exploration, while mind mapping functions as a cognitive scaffold that supports information processing, relationship organization, and decision-making within these contexts. This integration aligns closely with the constructivist principle of active knowledge construction. Preliminary studies have suggested that such an integrated teaching model may improve students’ cognitive abilities and learning outcomes (15, 16).

Guided by constructivist learning theory, the present study aimed to design and implement a structured integrated teaching model that systematically embeds mind mapping throughout the entire CBL process using simulated clinical cases. Mind mapping was employed as a core tool for cognitive organization and problem construction. In addition to examining improvements in students’ theoretical knowledge and practical nursing skills, this study placed particular emphasis on evaluating changes in cognitive and affective domains, including clinical reasoning, systems thinking, evidence-based decision-making, and learning motivation. Through this approach, the study seeks to provide a clear, evaluable, and evidence-based teaching framework for nursing education.

## Materials and methods

### Ethics statement

Ethical approval for the study was granted by the Ethics committee of Medical College Jinzhou Medical University, and informed consent was obtained from all participants.

### General information

One hundred undergraduate nursing students from Class 1 and Class 2 of the 2022 cohort at Medical College Jinzhou Medical University were enrolled in this study. Inclusion criteria were as follows: age above 20 years; full-time nursing majors admitted through the national unified entrance examination; completion of relevant theoretical courses; and provision of signed informed consent. Exclusion criteria included failure to complete the required theoretical courses or withdrawal from the program. Students were allocated to the research and control groups according to their original class assignment, with Class 1 serving as the research group and Class 2 as the control group (*n* = 50 per group). Based on published data (17), we assumed a normal distribution for test scores. The anticipated post-intervention theoretical test mean in the research group was 92.36 (SD = 4.45), while the control group mean was 87.36 (SD = 5.30). The significance level (α) was established at 0.05 (for a two-tailed test), and the test power (1−β) was set at 0.80. By applying the sample size formula n=2⁢(σ12+σ22)⁢(Z(1-α/2)+Z(1-β))2(μ1-μ2)2 for comparing two independent sample means and substituting the relevant parameters $n=\frac{2\,\left(4.45^{2}+5.30^{2}\right)\,{\left(1.96+0.84\right)}^{2}}{{\left(92.36-87.36\right)}^{2}}\approx 30$, the required sample size per group was determined. Previous literature (18) has demonstrated that the asymptotic relative efficiency of nonparametric tests (e.g., Mann-Whitney U test) relative to parametric tests (e.g., *t*-test) is approximately 95.5%. Consequently, the nonparametric sample size was calculated as $\frac{30}{0.955}\approx 31$. The actual enrollment of 50 participants per group met and exceeded the minimum statistical requirements.

### Methods

In the control group, instruction followed a conventional lecture-based teaching format. Course content was organized according to textbook chapters, with instructors presenting disease concepts, pathophysiology, nursing assessments, and standard nursing procedures using PowerPoint slides. Students primarily engaged in passive listening and note-taking and were assigned textbook-based exercises for after-class consolidation. During classroom teaching, instructors dominated the instructional pace and occasionally incorporated simple case analyses; however, interactive discussions were rarely organized due to limited class hours. For skills training sessions, a standardized process of “teacher demonstration–student group imitation–teacher round-robin error correction” was adopted, with emphasis placed on mastering basic nursing operational standards. Overall, teaching in the control group focused on procedural learning objectives, particularly the completeness of knowledge transmission and the accuracy of operational procedures. Assessment methods mainly consisted of final theoretical written examinations and practical skill evaluations, with continued emphasis on knowledge completeness and procedural accuracy.

In contrast, the research group received a mind mapping-integrated CBL model using simulated clinical cases. The overall instructional design emphasized the cultivation of cognitive learning objectives and guided students to develop logical reasoning, deep thinking, and clinical decision-making abilities through a multi-stage teaching process. Prior to class, instructors designed scaffolded learning tasks and required students to anchor their preparation around core disease entities. Through self-directed study of authoritative textbooks, nursing guidelines, and up-to-date literature, students constructed dynamic mind maps integrating etiology, pathophysiology, clinical manifestations, nursing assessment, diagnosis, planning, implementation, and evaluation. By leveraging the visual and logical structuring advantages of mind mapping, students were encouraged to organize knowledge relationships and derive key nursing priorities, rather than merely memorizing operational procedures. Preliminary peer evaluation and optimization were conducted within student groups, while instructors provided targeted feedback through an online platform to guide refinement of individual knowledge frameworks.

During in-class implementation, a “dual-teacher collaboration” model was adopted. Clinical nursing experts collaborated only with the teaching team to design highly simulated clinical cases, construct simulated ward scenarios, and embed unexpected situational developments, but did not participate in classroom teaching, skills training guidance, pre-class task assignment, or after-class tutoring. Students worked in teams, assuming roles such as primary nurse or team leader, and completed the full nursing process, including patient admission, assessment, individualized care planning, and emergency management. To enhance adaptability, instructors deliberately introduced sudden challenges, such as medication conflicts or questioning from family members. At the same time, the “5W1H” questioning method (Who, What, When, Where, Why, and How) was systematically applied within simulated clinical scenarios to guide layered inquiry. Students were prompted to consider why specific nursing measures were selected and their theoretical and clinical rationales; what constituted the core nursing problems and key monitoring indicators; when nursing interventions should be initiated or adjusted; where the critical sites or links of nursing operations were located; who should implement nursing measures and which departments required collaboration; and how nursing procedures should be standardized, optimized, and individualized. Through structured questioning across these six dimensions, students were guided to conduct full-chain nursing reasoning, promote deep reflection, and gradually construct a rigorous and comprehensive clinical nursing decision-making framework.

In the post-class extension phase, students were required to visualize their clinical reasoning processes by transforming decision pathways into flowcharts and submitting reflective reports incorporating standardized patient feedback. Instructors further enriched learning by delivering variant cases through a virtual simulation platform. This iterative cycle of “mind mapping–clinical practice–reflection and optimization” was designed to comprehensively enhance students’ critical thinking, teamwork, and clinical decision-making abilities.

In this study, the same core instructor was responsible for teaching both the research group and the control group. Total teaching duration, class-hour allocation, teaching objectives, and assessment criteria were fully consistent between the two groups.

### Evaluation criteria

Learning outcomes were assessed by comparing pre- and post-learning scores between the two groups. Theoretical performance was evaluated using a written examination with a maximum score of 100 points. Practical nursing skills were assessed through simulated operational tests randomly selected from core nursing skill sets, also scored on a 100-point scale. Higher scores indicated better mastery of theoretical knowledge and practical skills.

Changes in clinical thinking ability were evaluated with the validated Clinical Thinking Scale (19) pre- and post-learning, which covers three domains: critical thinking, evidence-based thinking, and systems thinking. Each student was independently assessed by two qualified faculty members, and the average of the two ratings was used for analysis. The scale contains 24 items rated on a five-point Likert scale (1 = very poor to 5 = excellent), with eight items per domain. Subscale scores range from 8 to 40, and higher scores indicate stronger clinical thinking ability.

Teaching effectiveness was measured with a structured questionnaire (20) pre- and post-learning, completed by instructors based on students’ classroom performance. This questionnaire includes three domains: active learning ability, learning enthusiasm (motivation), and clinical application ability. It consists of 24 items rated on a five-point Likert scale (1 = strongly disagree to 5 = strongly agree), with eight items per domain. Domain scores range from 8 to 40, with higher scores indicating greater teaching effectiveness.

After completion of the learning intervention, student satisfaction with the instructional model was assessed using a self-developed institutional questionnaire consisting of 20 items, each scored on a five-point scale, yielding a total possible score of 100. Satisfaction levels were classified as very satisfied (80–100), satisfied (60–79), and dissatisfied (<60). The overall satisfaction rate was calculated as (very satisfied + satisfied) ÷ total × 100%.

### Statistical analysis

Statistical analyses were performed using SPSS version 25.2 and GraphPad Prism version 10.0. The Shapiro–Wilk test was applied to assess data normality. Measurement data with a normal distribution were expressed as mean ± standard deviation, whereas data with a non-normal distribution were presented as median (P25, P75). The Wilcoxon signed-rank test was used to compare pre- and post-intervention data within groups. To evaluate the effect of the intervention on learning gains, change values (post-test minus pre-test) were calculated for all primary outcome indicators, and differences between the two groups were compared using the Mann–Whitney U test. Categorical data were expressed as percentages (%) and compared between groups using the χ^2^ test. To assess the practical significance of observed differences, effect sizes (*r*-values) were calculated for between-group comparisons, where *r* = 0.1–0.3 indicates a small effect, 0.3–0.5 indicates a medium effect, and >0.5 indicates a large effect. The significance level was set at α = 0.05, with *P* < 0.05 considered statistically significant. For multiple comparisons, Bonferroni correction was applied, and the adjusted significance level (α′) was used to determine statistical significance.

## Results

### General information

No significant differences were observed between the two cohorts with respect to gender, age, educational background, pre-study assessment scores, or clinical thinking ability (*P* > 0.05) (Table 1).

**TABLE 1:** Comparison of general information between the two groups [*n* (%)]/M (P25, P75).

General information	Research group (*n* = 50)	Control group (*n* = 50)	χ ^2^/*z*	*P*
Gender			0.088	0.766
Male	7 (14.00)	6 (12.00)		
Female	43 (86.00)	44 (88.00)		
Age (year)	24.00 (23.00, 24.00)	23.00 (22.00, 24.00)	1.271	0.204
Educational background			0.071	0.790
College entrance examination admission	41 (82.00)	42 (84.00)		
Preparatory class	9 (18.00)	8 (16.00)		
Main indicators (pre-study)				
Theoretical assessment score	75.00 (68.00, 84.00)	77.50 (69.00, 82.00)	0.317	0.751
Nursing skills assessment score	75.50 (70.00, 83.00)	74.50 (65.00, 82.00)	1.146	0.252
Critical thinking ability	15.00 (12.00, 20.00)	14.50 (10.00, 18.00)	1.383	0.167
Evidence-based thinking ability	14.50 (10.00, 19.00)	14.00 (11.00, 19.00)	0.021	0.983
Systematic thinking ability	17.50 (11.00, 20.00)	15.00 (13.00, 18.00)	0.778	0.437
Active learning ability	17.00 (13.00, 21.00)	15.00 (12.00, 18.00)	1.193	0.233
Learning enthusiasm (motivation)	14.00 (12.00, 19.00)	14.00 (10.00, 18.00)	0.363	0.717
Clinical application ability	15.00 (12.00, 19.00)	16.00 (12.00, 19.00)	0.014	0.989

### Theoretical knowledge and nursing skills

At baseline, the two groups demonstrated comparable performance in both theoretical examinations and nursing skill assessments (*P* > 0.05). Following the learning period, both groups experienced a substantial rise in these scores compared to their pre-learning levels (*P* < 0.05). Moreover, the research group outperformed the control group, achieving markedly higher scores (*z* = 4.358, and 7.680; *P* < 0.05).

To further clarify the impact of the intervention on learning gains, score change values (post-test minus pre-test) were calculated for both groups and compared using the Mann–Whitney U test. The median learning gain in theoretical assessment was 17.00 points in the research group and 9.50 points in the control group, with a significant inter-group difference (*Z* = −2.583, *P* = 0.011, *r* = 0.26, indicating a small-to-medium effect). For nursing skills assessment, the median learning gain was 18.50 points in the research group compared with 6.50 points in the control group, also showing a significant inter-group difference (*Z* = −4.339, *P* < 0.001, *r* = 0.43, indicating a medium effect) (Table 2).

**TABLE 2:** Comparison of theoretical assessment scores and nursing skills assessment scores between the two groups (point).

Group		Research group (*n* = 50)	Control group (*n* = 50)	*z*	*P*	Effect size (r)
Theoretical assessment scores	Before learning	75.00 (68.00, 84.00)	77.50 (69.00, 82.00)	0.317	0.751	
	After learning	92.00 (89.00, 96.00)*	87.00 (83.00, 92.00)*	4.358	<0.001	
Learning gain (change value)		17.00	9.50	−2.583	0.011	0.26
Nursing skills assessment scores	Before learning	75.50 (70.00, 83.00)	74.50 (65.00, 82.00)	1.146	0.252	
	After learning	94.00 (90.00, 98.00)*	81.00 (77.00, 88.00)*	7.680	<0.001	
Learning gain (change value)		18.50	6.50	−4.339	<0.001	0.43

*Compared with the same group before learning, *P* < 0.05. The *z* and *P*-values for learning gains are the results of the Mann-Whitney U test; the effect size *r* = Z/√ (n_1_ + n_2_), where *r* = 0.1–0.3 indicates a small effect, 0.3–0.5 indicates a medium effect, and >0.5 indicates a large effect.

### Clinical thinking ability

Before learning, there were no significant group differences in critical thinking, evidence-based thinking, or systems thinking (*P* > 0.05). Post-learning, both cohorts exhibited significant gains across all three domains (*P* < 0.05). The research group showed superior improvements, with noticeably higher scores than the control group (*z* = 4.149, 4.525, and 4.181; *P* < 0.05).

Further comparison of inter-group differences in learning gains (post-test minus pre-test) using the Mann-Whitney U test revealed significant differences between the two groups in critical thinking ability, evidence-based thinking ability, and systematic thinking ability (*z* = −2.688, −3.572, and −2.665; *r* = 0.27, 0.36, and 0.27, respectively) (Table 3).

**TABLE 3:** Comparison of clinical thinking abilities between the two groups (point).

Group		Research group (*n* = 50)	Control group (*n* = 50)	*z*	*P*	Effect size (r)
Critical thinking	Before learning	15.00 (12.00, 20.00)	14.50 (10.00, 18.00)	1.383	0.167	
	After learning	33.50 (29.00, 38.00)*	30.50 (23.00, 32.00)*	4.149	<0.001	
Learning gain (change value)		18.50	16.00	−2.688	0.007	0.27
Evidence-based thinking	Before learning	14.50 (10.00, 19.00)	14.00 (11.00, 19.00)	0.021	0.983	
	After learning	32.00 (29.00, 35.00)*	27.00 (24.00, 33.00)*	4.525	<0.001	
Learning gain (change value)		17.50	13.00	−3.572	<0.001	0.36
Systems thinking	Before learning	17.50 (11.00, 20.00)	15.00 (13.00, 18.00)	0.778	0.437	
	After learning	33.00 (29.00, 35.00)*	29.00 (23.00, 32.00)*	4.181	<0.001	
Learning gain (change value)		15.50	14.00	−2.665	0.008	0.27

*Compared with the same group before learning, *P* < 0.05. The *z* and *P*-values for learning gains are the results of the Mann-Whitney U test; the effect size *r* = Z/√ (n_1_ + n_2_), where *r* = 0.1–0.3 indicates a small effect, 0.3–0.5 indicates a medium effect, and >0.5 indicates a large effect.

### Teaching effectiveness

At baseline, no significant differences were observed between the two groups in active learning, enthusiasm, or clinical application capacity (*P* > 0.05). Post-learning, the research group achieved significantly higher scores across all three dimensions compared with the control group (*z* = 4.651, 5.396, and 6.032, respectively; *P* < 0.05).

Further analysis of inter-group differences in learning gains (post-test minus pre-test) using the Mann-Whitney U test revealed significant differences between the two groups in active learning ability, learning enthusiasm (motivation), and clinical application ability (*z* = −3.011, −3.959, and −4.550, respectively) (Table 4).

**TABLE 4:** Comparison of teaching effectiveness between the two groups (point).

Group		Research group (*n* = 50)	Control group (*n* = 50)	*z*	*P*	Effect size (r)
Self-directed learning ability	Before learning	17.00 (13.00, 21.00)	15.00 (12.00, 18.00)	1.193	0.233	
	After learning	32.00 (28.00, 35.00)*	26.50 (23.00, 31.00)*	4.651	<0.001	
Learning gain (change value)		15.00	11.50	−3.011	0.003	0.30
Learning enthusiasm	Before learning	14.00 (12.00, 19.00)	14.00 (10.00, 18.00)	0.363	0.717	
	After learning	33.00 (29.00, 37.00)*	27.00 (24.00, 30.00)*	5.396	<0.001	
Learning gain (change value)		19.00	13.00	−3.959	<0.001	0.40
Clinical application skills	Before learning	15.00 (12.00, 19.00)	16.00 (12.00, 19.00)	0.014	0.989	
	After learning	34.00 (30.00, 37.00)*	27.00 (24.00, 31.00)*	6.032	<0.001	
Learning gain (change value)		19.00	11.00	−4.550	0.000	0.46

*Compared with the same group before learning, *P* < 0.05. The *z* and *P*-values for learning gains are the results of the Mann-Whitney U test; the effect size *r* = Z/√ (n_1_ + n_2_), where *r* = 0.1–0.3 indicates a small effect, 0.3–0.5 indicates a medium effect, and >0.5 indicates a large effect.

### Teaching satisfaction

In the research group, 49 students (98.0%) reported being satisfied with the teaching approach, compared with 40 students (80.0%) in the control group. The overall satisfaction rate was significantly higher in the research group than in the control group (*χ^2^* = 8.274, *P* < 0.05) (Table 5).

**TABLE 5:** Comparison of teaching satisfaction rate between the two groups (%).

Group	*n*	Very satisfied	Satisfied	Dissatisfied	Satisfaction rate
Research group	50	31 (62.00)	18 (36.00)	1 (2.00)	49 (98.00)
Control group	50	27 (54.00)	13 (26.00)	10 (20.00)	40 (80.00)
*χ^2^*					8.274
*P*					0.004

## Discussion

This study demonstrates that integrating mind mapping with CBL in simulated clinical thinking scenarios significantly enhances nursing students’ academic performance, clinical competencies, and learning satisfaction compared with conventional lecture-based instruction. These findings confirm the potential value of a composite, student-centered teaching strategy that combines visual cognitive tools with situated inquiry-based learning (16).

First, the significant advantages observed in the research group with respect to both theoretical knowledge and nursing skills assessments support the effectiveness of this integrated model in promoting knowledge integration and practical application. This finding is consistent with conclusions reported in previous studies within the field of nursing education. For example, the combination of concept mapping with simulated clinical cases has been shown to significantly enhance students’ ability to apply and integrate knowledge in complex clinical contexts (21). In nursing education, this approach facilitates the transformation of theoretical knowledge into practical clinical reasoning (21). Similarly, in medical students’ pathophysiology education, the incorporation of simulated concept map exercises has been reported to strengthen understanding and application of complex mechanisms (22). In ultrasound medicine training, residents who adopted a “CBL + mind mapping” model also achieved significantly higher scores in both theoretical examinations and clinical operational assessments compared with those receiving traditional lecture-based instruction (23).

Second, the marked improvements in critical thinking, evidence-based thinking, and systems thinking observed in this study carry important educational implications. These higher-order cognitive abilities are central to contemporary nursing practice and play a critical role in clinical decision-making and patient safety. Previous research has demonstrated that integrating mind mapping into nursing professional courses can effectively enhance nursing students’ critical thinking abilities (10). In addition, studies focusing on student nurses have shown that CBL significantly improves critical thinking in clinical decision-making, particularly in the domains of analysis, evaluation, and deductive reasoning (24). The integrated teaching model employed in this study may promote such cognitive advancement through multiple mechanisms. Specifically, the uncertainty and complexity inherent in simulated clinical cases encourage students to engage in continuous judgment and decision-making, while mind maps function as cognitive scaffolds that externalize and structure these implicit reasoning processes. This combination guides students to systematically evaluate evidence, analyze problems, and construct comprehensive solutions.

In addition, the superior performance of the research group in active learning ability, learning enthusiasm, and teaching satisfaction provides strong support for the sustainability and learner acceptance of this instructional model. These outcomes align with the positive affective and motivational effects anticipated by student-centered educational philosophies. Prior research has confirmed that instructional designs integrating online interaction with face-to-face case discussions can significantly enhance learners’ autonomy and engagement, ultimately leading to higher satisfaction levels (25, 26). By combining the visual exploration afforded by mind mapping with the collaborative inquiry emphasized in CBL, the present model may better satisfy learners’ intrinsic psychological needs for autonomy (through self-construction of knowledge maps), competence (through successful resolution of clinical cases), and relatedness (through teamwork and collaboration). This process may foster more enduring intrinsic motivation. Such positive learning experiences not only contribute to immediate instructional effectiveness but may also exert a lasting influence on nursing students’ professional identity formation and commitment to lifelong learning. A prior study has similarly reported that CBL broadens teaching perspectives and stimulates enthusiasm, thereby strengthening learners’ autonomy and yielding favorable outcomes (2). Likewise, mind mapping techniques have been shown to improve teaching efficiency and increase student satisfaction, underscoring their value in optimizing educational resources (27, 28). Conversely, the relatively lower satisfaction observed in the control group highlights the limitations of traditional, teacher-centered instructional models, which often fail to meet contemporary students’ needs for active engagement and clinical relevance.

Based on the findings of this study, mind mapping facilitates the integration of fragmented information into a coherent cognitive framework through non-linear visual dual coding, thereby enhancing deep information processing and long-term knowledge retention (19, 20). In parallel, CBL promotes students’ analytical reasoning and clinical decision-making abilities by guiding them to apply theoretical knowledge through iterative analysis of simulated cases (23). The integration of these two approaches establishes a complementary mechanism that supports knowledge integration and clinical problem-solving. Specifically, this model clarifies the instructional logic of systematically embedding mind maps as cognitive scaffolding within the CBL process, offering a more detailed mechanistic explanation of how these strategies jointly reduce cognitive load and promote meaningful learning. From a curriculum design perspective, the modular structure of a “simulated case–driven, thinking tool–supported” approach demonstrates transferability and may serve as a reference framework for similar curriculum reforms. Theoretically, this integrated model aligns with constructivist learning theory, cognitive load theory, and the experiential learning cycle, providing a plausible conceptual framework for interpreting its educational effects. In terms of teaching practice, effective implementation of this model depends on appropriate faculty development and training to support instructors’ transition from knowledge transmitters to learning designers. At a broader level, the findings provide preliminary empirical support for curriculum and assessment reforms oriented toward the cultivation of core cognitive competencies. Nevertheless, more systematic impacts–such as sustained institutional policy changes or resource reallocation–require further validation through long-term, multi-context investigations.

Caution is warranted when interpreting the observed improvements. In addition to the potential cognitive benefits of the intervention itself, alternative explanations should be considered. Students in the research group may have demonstrated heightened attention due to the novelty of the instructional approach, resulting in a potential “novelty effect.” Moreover, increased instructor enthusiasm associated with implementing an innovative teaching model may have acted as a latent variable influencing student motivation and performance. The possibility of a “Hawthorne effect,” whereby participants modify behavior due to awareness of being studied, also cannot be entirely excluded. Collectively, these factors suggest that some observed gains–particularly in subjective outcomes such as learning enthusiasm and satisfaction–may partially reflect sociopsychological dynamics rather than purely cognitive intervention effects. This consideration does not diminish the value of the intervention but underscores the need for more rigorous attribution of outcomes through methods such as blinded assessments, long-term follow-up, and qualitative inquiry in future research.

Despite these promising findings, several limitations should be noted. First, this study employed a single-center design with student grouping based on natural classes, and some evaluation instruments (such as the satisfaction questionnaire) were self-developed and lacked formal validation. These factors may introduce selection bias, measurement error, and constraints on the generalizability of the findings. Second, the inclusion of a dual-instructor model and the potentially higher level of instructor engagement in the experimental condition, although enhancing instructional authenticity, may have introduced confounding influences affecting internal validity. Finally, the outcomes assessed in this study reflected short-term effects, leaving unanswered questions regarding long-term knowledge retention and the transfer of skills to authentic clinical practice.

In conclusion, the results of this study indicate that implementing a mind mapping–integrated CBL model using simulated clinical cases in nursing education can effectively enhance students’ theoretical knowledge, clinical operational skills, higher-order cognitive abilities (including critical thinking, evidence-based thinking, and systems thinking), and learning satisfaction. Overall, this integrated approach demonstrates superior instructional effectiveness compared with traditional lecture-based teaching. These findings support ongoing reforms in nursing education that emphasize the integration of visual cognitive tools with situated problem-solving. Future research should advance in several directions. First, multi-center randomized controlled studies with extended follow-up periods–such as tracking outcomes into clinical internships–are needed, alongside the use of standardized assessment tools with established reliability and validity, to strengthen the evidence base. Second, systematic investigation of the conditions necessary for successful implementation of this model is warranted, particularly with regard to faculty training and professional development frameworks that support instructors’ transition from lecturers to learning designers, thereby laying the groundwork for sustainable and scalable institutional adoption.

## Data Availability

The raw data supporting the conclusions of this article will be made available by the authors, without undue reservation.
